# Model-based assessment of combination therapies – ranking of radiosensitizing agents in oncology

**DOI:** 10.1186/s12885-023-10899-y

**Published:** 2023-05-06

**Authors:** Marcus Baaz, Tim Cardilin, Floriane Lignet, Astrid Zimmermann, Samer El Bawab, Johan Gabrielsson, Mats Jirstrand

**Affiliations:** 1 Fraunhofer-Chalmers Research Centre for Industrial Mathematics, Gothenburg, Sweden; 2 Department of Mathematical Sciences, Chalmers University of Technology and University of Gothenburg, Gothenburg, Sweden; 3 Translational Medicine, Quantitative Pharmacology, Merck Healthcare KGaA, Darmstadt, Germany; 4 Translation Innovation Platform Oncology, Merck Healthcare KGaA, Darmstadt, Germany; 5 Present Address: Translational Medicine, Servier, Suresnes, France; 6 Meddoor AB, Gothenburg, Sweden

**Keywords:** Radiation therapy, Combination therapy, Tumor static exposure, Non-linear mixed effects, Inter-study variability

## Abstract

**Background:**

To increase the chances of finding efficacious anticancer drugs, improve development times and reduce costs, it is of interest to rank test compounds based on their potential for human use as early as possible in the drug development process. In this paper, we present a method for ranking radiosensitizers using preclinical data.

**Methods:**

We used data from three xenograft mice studies to calibrate a model that accounts for radiation treatment combined with radiosensitizers. A nonlinear mixed effects approach was utilized where between-subject variability and inter-study variability were considered. Using the calibrated model, we ranked three different Ataxia telangiectasia-mutated inhibitors in terms of anticancer activity. The ranking was based on the Tumor Static Exposure (TSE) concept and primarily illustrated through TSE-curves.

**Results:**

The model described data well and the predicted number of eradicated tumors was in good agreement with experimental data. The efficacy of the radiosensitizers was evaluated for the median individual and the 95% population percentile. Simulations predicted that a total dose of 220 Gy (5 radiation sessions a week for 6 weeks) was required for 95% of tumors to be eradicated when radiation was given alone. When radiation was combined with doses that achieved at least 8 $\mu \mathrm{g}/\mathrm{mL}$ of each radiosensitizer in mouse blood, it was predicted that the radiation dose could be decreased to 50, 65, and 100 Gy, respectively, while maintaining 95% eradication.

**Conclusions:**

A simulation-based method for calculating TSE-curves was developed, which provides more accurate predictions of tumor eradication than earlier, analytically derived, TSE-curves. The tool we present can potentially be used for radiosensitizer selection before proceeding to subsequent phases of the drug discovery and development process.

## Background

Radiosensitizers are given in combination with radiation treatment to make tumor tissue more sensitive to radiation [1]. Hypoxic cells are more resistant to radiation treatment and an important class of radiosensitizers, Oxygen Mimetics, function by reducing the number of them [2]. Another important class of radiosensitizers is those that regulate important pathways, such as inhibitors of enzymes involved in the DNA repair mechanism for single- and double-stranded breaks [3]. Examples of such radiosensitizers are Ataxia telangiectasia-mutated (ATM) inhibitors. ATM is an important serine/threonine kinase in the body’s DNA damage repair system and as the name implies, the radiosensitizers work by inhibiting this kinase [3, 4]. New possibilities have arisen in the development of radiosensitizers thanks to the advent of nanotechnology. High Z (atomic number) heavy-metal nanomaterials, such as gold and silver particles, have been shown to be promising radiosensitizers because of their ability to absorb, scatter, and emit radiation energy [5, 6]. Moreover, the physical properties of these particles can be tailored to, e.g., increase the accumulation at the tumor target site [7]. With the help of radiosensitizers, the radiation dose can thus be adjusted to maximize anticancer efficacy while reducing harmful side effects. However, despite showing promising results in preclinical studies, only about 15% of all novel anticancer drugs that enter clinical trials eventually gain regulatory approval [8].

Both preclinical and clinical results in oncology [9, 10] can be hard to replicate and commonly cited factors are poor experimental design and faulty use of statistical tools [11]. Another contributing factor is large inter-study variability. Inter-study variability can be caused by, e.g., experiments being carried out at different times of the day and differences in experimental conditions for sampling [12, 13]. Therefore, when evaluating the efficacy of a test compound, it is important to take these limitations into consideration. Doing so allows for more robust model predictions, increasing the chances that findings can be successfully replicated. Furthermore, more robust preclinical model predictions would also help design better clinical studies to test efficacy in humans.

Mathematical modeling is a powerful tool that can support preclinical drug discovery and development in oncology [14, 15]. Using, for example, human tumor xenograft data, one can evaluate the efficacy of an anticancer compound and make predictions of its potential for clinical use [16]. Mathematical modeling is also useful for ranking test compounds based on anticancer efficacy and toxicity [17, 18] as well as predicting optimal dose levels or treatment schedules [19–21]. Quantitative techniques are particularly useful for analyzing combination therapies since all possible combinations of doses and treatment agents cannot be tested experimentally [22]. Several models for both preclinical and human data have been developed to describe the effects of radiation treatment [23–25] as well as combination therapies [26–30].

One is often interested in finding a threshold value for the exposure of a compound, that when exceeded results in the desired treatment outcome, e.g., tumor shrinkage or eradication [27, 31–33]. One such threshold value is the so-called Tumor Static Exposure (TSE), sometimes referred to as Tumor Static Concentration. TSE is defined as all combinations of exposure levels that if kept constant result in tumor stasis and therefore separate the space of all possible exposures into a region of tumor growth and a region of tumor shrinkage [26, 34]. Administering combinations of test compounds and radiation yielding an exposure above the TSE-curve is predicted to lead to tumor shrinkage and eventually tumor eradication. Analytical expressions for TSE are typically found from the mathematical model describing the anti-tumor activity of the investigated compounds. One of the benefits of this is that one can immediately read from such an expression how different model parameters affect the TSE [28]. However, when complex models describe the anti-tumor activity, it may not be possible to derive analytical expressions for TSE without simplifications. One way of getting around this problem is that instead of finding an analytical expression for TSE, one can resort to numerical methods and simulations.

This paper has three aims: (i) Investigate tumor volume data from three xenograft studies where radiation treatment (here with external photon beam) in combination with three different ATM inhibitors was tested. To accomplish this, we fit and evaluate a nonlinear mixed effects (NLME) model to the data [35]. Both between-subject variability and inter-study variability are considered. (ii) Develop a simulation-based method for calculating the TSE for complicated tumor growth models and evaluate the tumor eradication predictions from this method with those of an analytically derived TSE expression. (iii) Use TSE to rank the three radiosensitizers based on their anticancer efficacy.

## Methods

### Experimental data

Data from three different studies were jointly analyzed in this paper. All three studies were carried out to investigate the efficacy of three radiosensitizers, which we denote by $R{s}_{1}$, $R{s}_{2}$, and $R{s}_{3}$.

$R{s}_{1}$ was used in all three studies, whereas the other two radiosensitizers were only present in study 1. An overview of the three studies is given in Table 1. Part of the data from study 1 has previously been published and used for modeling [35, 36]. Data from study 2 have also previously been published and used for modeling but, with a different radiation model [37]. Data from study 3 have not been previously published. The experimental conditions were the same for all three studies and have been described previously in [29, 30]. The conditions are repeated below with minor additions to the text.

**Table 1:** The study number, sample size, duration, treatment groups, and PK sampling times for each of the three xenograft studies used in this paper

Study	Sample size^a^	Duration of treatment	Treatment Groups (listed treatment applied daily for 5 days a week)	PK sampling times^b^
1	9	6 weeks	Vehicle, 2 Gy Radiation 100 mg/kg $R{s}_{1}$+ 2 Gy Radiation 25 mg/kg and 100 mg/kg $R{s}_{2}$+ 2 Gy Radiation 20 mg/kg $R{s}_{3}$+ 2 Gy Radiation	2, 4, and 6 h
2	10	1 week	Vehicle, 2 Gy Radiation 10, 50 and 200 mg/kg $R{s}_{1}$	0.5, 2, 4, 6, and 24 h
3	10	6 weeks	2 Gy Radiation 25, 50 and 100 mg/kg $R{s}_{1}$ + 2 Gy Radiation	2, 4, and 6 h

^a^Per treatment group

^b^After a single dose

The study designs and animal usage for the xenograft studies were approved by local animal welfare authorities (Regierungspräsidium Darmstadt, Germany, protocol numbers DA4/Anz. 397, DA4/Anz. 398, and DA4/Anz.1014). Female 7–9-week-old CD1 or NMRI nude mice were purchased from Charles River Laboratories (Sulzfeld, Germany) and allowed to adapt to local housing conditions for at least 1 week. All animals were implanted with electronical transponders to enable individual identification. Tumors were grafted upon subcutaneous injections of 2.5 × 106 cultured FaDu (ATCC HBT-43, human pharyngeal squamous carcinoma) cells in the flank or thigh (radiotherapy with total doses > 10 Gy). Cells were injected in 100 µl PBS/Matrigel (BD MatrigelTM Matrix) (1:1) (thigh) or PBS only (flank). Tumor sizes were measured with electronic calipers twice weekly. Length (L) was measured along the longest axis of the tumor and width (W) was measured perpendicular to the length. Volumes were calculated using the equation L × W^2/2. Mice with established xenografts were randomized (typical sample size *n* = 9–10 from 15 mice/arm) to obtain a similar mean and median within nonblinded treatment groups (average starting volume ~ 100 mm3).

Mice were treated on the same or the following day after randomization. Test agents were suspended in vehicle of 0.5% Methocel K4M, 0.25% Tween20, 300 mM sodium citrate buffer pH 3.2, or 100 mM sodium citrate buffer pH 4.5, and given by oral gavage in a volume of 10 ml/kg,10 min before each irradiation (IR) fraction. IR was administered locally by positioning the tumor-bearing area under the beam while shielding the rest of the body with a lead shield. Mice were anesthetized and irradiated in groups of 9 or 10 with 2 Gy/fraction using the same X-RAD320 cabinet (Precision X-ray Inc.) set to 10 mA, 250 kV, 58 s, 50 cm FSD collimator, 2 mm A1 filter with the same voltage and filters on the tubes.

Animals were euthanized at the end of the experiment by either exposition to CO_2_, or overdosing on narcotic agents (ip application of Ketamine (160 mg/kg)/Rompune (75 mg/kg), or Ketamine (75 mg/kg)/Medetomidine (1 mg/kg)) and subsequent axillary cut.

#### Pharmacokinetic and pharmacodynamic data

In study 1, mice were given radiation and radiosensitizer treatment 5 days a week for 6 weeks. The study contained the following treatment groups; vehicle, 2 Gy radiation, and 2 Gy radiation in combination with either 100 mg/kg $R{s}_{1}$, 25 mg/kg $R{s}_{2}$, 100 mg/kg $R{s}_{2}$, or 20 mg/kg $R{s}_{3}$. Efficacy was assessed twice a week (sample size 9) and plasma was sampled for pharmacokinetics (PK) evaluation in additional animals 2, 4, and 6 h after the first dose.

Study 2 was a shorter study where the mice were treated 5 times for 1 week. Tumor volumes were, however, still recorded for 120 days for the highest dose. The study contained the following treatment groups; 2 Gy radiation and 2 Gy radiation in combination with either 25 mg/kg, 50 mg/kg, or 100 mg/kg $R{s}_{1}$. Efficacy was assessed twice a week (sample size 10) and PK was sampled after 0.5, 2, 4, 6, and 24 h after the first dose.

In study 3, mice were given radiation and radiosensitizer treatment 5 days a week for 6 weeks. This study contained 4 treatment groups, 2 Gy radiation and 2 Gy radiation in combination with either 25 mg/kg, 50 mg/kg, or 100 mg/kg $R{s}_{1}$. Efficacy was assessed twice a week (sample size 10) and PK was sampled 2, 4, and 6 h after the dose was given on days 1 and 8. A higher pH of buffer was used for $R{s}_{1}$ in study 1 compared to the other two studies.

### Exposure to radiosensitizer

The pharmacodynamic model is driven by a single pharmacokinetic value per radiation application. We tested using the exposure of the radiosensitizers at the instance of each radiation application as well as the average exposure of the radiosensitizers during treatment. Both PK models and direct reading of the observed maximum concentration, averaged over all individuals in the same dose groups, ${C}_{max}$, were used in the model building process.

### Pharmacodynamics

**Fig. 1:**
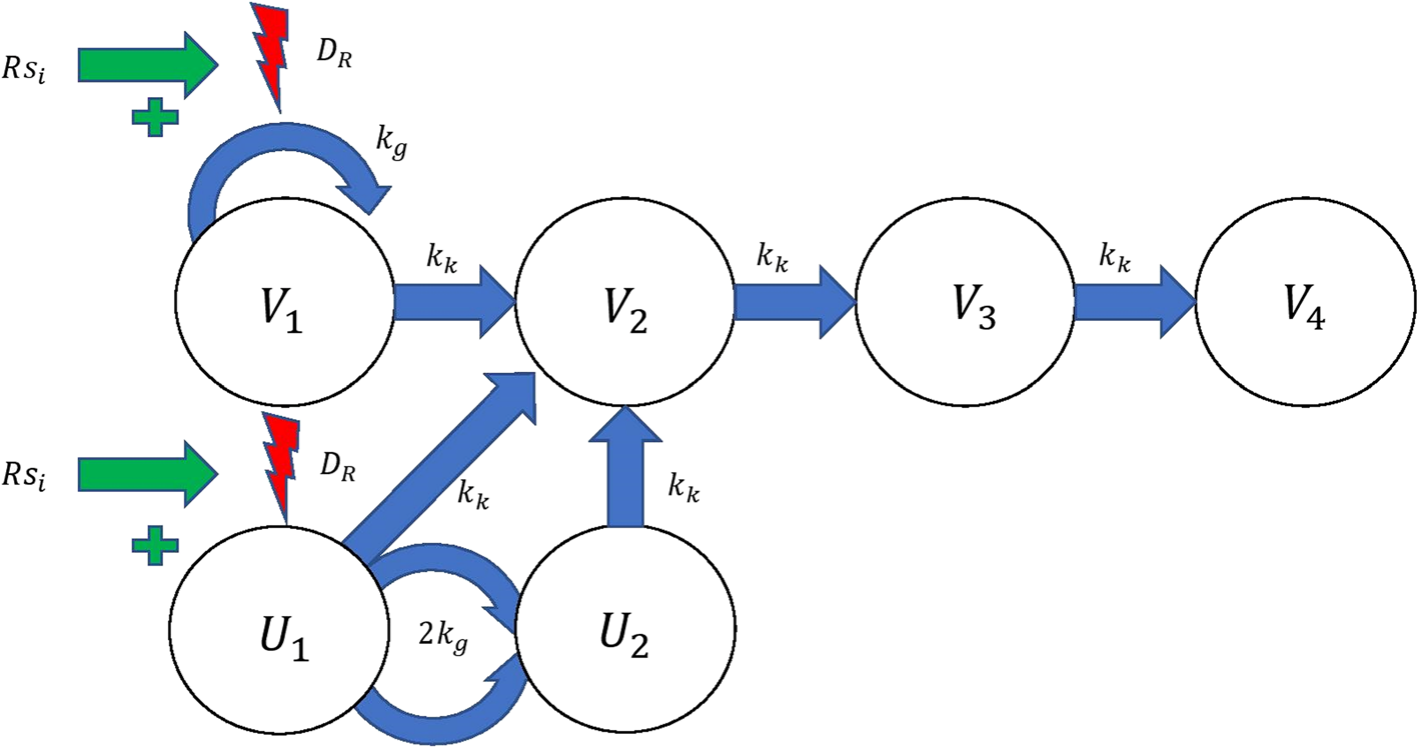
A schematic representation of the long-term radiation and radiosensitizer model. ${V}_{1}$ consists of proliferating tumor cells.${V}_{2}$, ${V}_{3}$, and ${V}_{4}$ are transit compartments consisting of damaged tumor cells. The growth rate of the proliferating cells is denoted by ${k}_{g}$ and the rate of natural cell death of all cells by ${k}_{k}$. As a result of the radiation treatment cells become radiation damaged and this affects them in two ways. Firstly, the growth rate is inhibited and secondly, a fraction of proliferating cells is irreversibly damaged at each radiation session. These damaged cells are moved to ${U}_{1}$ and can go through mitosis once, but their daughter cells (${U}_{2}$) cannot. Therefore, these radiation damage cells eventually also die. Both radiation effects are stimulated by the radiosensitizers. ${D}_{R}$ denotes the radiation dose and $R{s}_{i}$ denotes the exposure of radiosensitizer *i* at the instant of radiation application

To quantify the long-term effects of radiation combined with radiosensitizer, a model previously developed by Cardilin et al. was utilized [35]. In the model, the total tumor volume is assumed to be divided into three different types of compartments: proliferating cells, dying cells, and radiation damaged cells. A schematic representation of the model is shown in Fig. 1.

#### Tumor growth

Proliferating tumor cells are located in the first compartment ${V}_{1}$ and before the treatment start, these cells exhibit exponential growth at a rate equal to the difference between the growth rate,${k}_{g}$, and the natural cell death rate$, {k}_{k}$. As proliferating cells start to succumb to natural cell death, they undergo three stages of degradation before dying. These stages are represented by three transit compartments${V}_{2}$,${V}_{3}$, and${V}_{4}$, and once this process has started the cells lose the ability to proliferate. The number of transit compartments is chosen based on previous publications [35, 38]. Differentiating between proliferating and non-proliferating cells allows for a more biologically reasonable model and accounts for a delayed treatment response [31].

#### Long-term effect of radiation

The model assumes that the radiation treatment has two effects on proliferating cells: long–term and short-term. The long-term effect is described as a reduction of the growth rate based on the accumulated radiation dose used during treatment. This reflects the reduced growth rate after treatment that is observed in the data. Such phenomenon has been observed in other studies as well and may be explained by mutations and reduced vascularization in the tumor, as well as changes in the tumor microenvironment [39]. Accounting only for the long-term effect and assuming that the tumors are irradiated with the same radiation dose each time, ${D}_{R}$, turnover of proliferating cells after *n* radiation applications is described by the following equation,

1 $$\begin{array}{c}{D}_{Acc}=n {D}_{R},\\ \frac{d{V}_{1}}{dt}=\left({k}_{g}I\left({D}_{Acc}\right)- {k}_{k}\right){V}_{1}, t \ne {t}_{i}\hspace{2mm} i=1\dots n,\end{array}$$

Radiation applications occur at ${t}_{i}$, ${D}_{Acc}$ is the accumulated radiation dose, and *I*(${D}_{Acc}$) is an inhibitory function depending on the accumulated radiation dose. In earlier work [35], the inhibition was described by an exponential function, whereas we test three different functions to determine best fit with data. The three functions are: (i) an exponential function, (ii) a saturation function, and (iii) a linear function,

2 $$\begin{array}{c}I\left({D}_{Acc}\right)={e}^{-\gamma {D}_{Acc}}\\ I\left({D}_{Acc}\right)=1-\frac{\gamma {D}_{Acc}}{I{D}_{50}+{D}_{Acc}}\\ I\left({D}_{Acc}\right)=1-\gamma {D}_{Acc}\end{array}$$

where $\gamma$ is a parameter associated with the degree of inhibition of each function and $I{D}_{50}$ is the radiation dose that gives the half-maximal response.

#### Short-term effect of radiation

The most critical damage from radiation is double-stranded DNA breaks, although single-stranded DNA breaks can also occur [40]. The DNA strand breaks lead to apoptosis and mitotic catastrophe, which are thought to be two of the primary responses of tumor cells to irradiation [41]. These and other death mechanisms are accounted for in the model by a short-term radiation effect. This effect is described by a proportion of the proliferating cells being instantaneously damaged at each radiation application. The proportion of cells that are damaged is determined through the so-called linear-quadratic equation, given by,

3 $$F\left({D}_{R}\right)=1-{e}^{-\left(\alpha {D}_{R }+ \beta { D}_{R}^{2}\right)},$$

where $\alpha$ and $\beta$ are radiosensitivity parameters. This equation is commonly used in radiobiology to quantify cell death due to radiation treatment [40]. Turnover of proliferating cells at each radiation application can be described by

4 $${V}_{1}({t}_{i}^{+})={V}_{1}({t}_{i}^{-})-F\left({D}_{R}\right){V}_{1}\left({t}_{i}\right) i=1, \dots , n,$$

where ${t}_{i}^{-}$ and ${t}_{i}^{+}$ denotes the time immediately before and after each radiation application, respectively. The proliferating cells that suffer this kind of irreversible radiation damage are instantaneously moved from compartment ${V}_{1}$ to ${U}_{1}$. These cells are then allowed to go through mitosis once and for each cell that does, two daughter cells enter compartment ${U}_{2}$. As a result of the radiation these daughter cells cannot go through mitosis, but instead enters the chain of damaged compartments, ${V}_{2}$, ${V}_{3}$, and ${V}_{4}$, eventually leading to death. The compartments ${U}_{1}$ and ${U}_{2}$ are included to describe a delayed treatment response that agrees with the observation that irradiated cells can survive one or a few cell cycles before dying [42]. Turnover of radiation damaged cells is described by the equations,

5 $$\begin{array}{c}\frac{d{U}_{1}}{dt}=-{k}_{g}{U}_{1}-{k}_{k}{U}_{1}, t \ne {t}_{i}, \\ {U}_{1}\left({t}_{i}^{+}\right)={U}_{1}\left({t}_{i}^{-}\right)+F\left({D}_{R}\right){V}_{1}, i=1, \dots , n,\\ \frac{d{U}_{2}}{dt}=2{k}_{g}{U}_{1}-{k}_{k}{U}_{2}\end{array}$$

Note that as one cell in ${U}_{1}$ undergoes mitosis, two daughter cells enter ${U}_{2}$, hence the additional factor 2 in the equation for ${U}_{2}$.

#### Damaged cells and initial conditions

Turnover of damaged cells is described by the following set of equations,

6 $$\begin{array}{l} \frac{d{V}_{2}}{dt}={k}_{k}{V}_{1}+{k}_{k}{U}_{1}+{k}_{k}{U}_{2}-{k}_{k}{V}_{2}\\ \frac{d{V}_{3}}{dt}={k}_{k}{V}_{2}-{k}_{k}{V}_{3}\\ \frac{d{V}_{4}}{dt}={k}_{k}{V}_{3}-{k}_{k}{V}_{4}\end{array}$$

The initial conditions for the system of differential Eqs. 1, 5, and 6 are

7 $$\begin{array}{c}{V}_{i}\left(0\right)={V}_{0}{\left(\frac{{k}_{k}}{{k}_{g}}\right)}^{i-1} i=1, 2, 3, 4,\\ {U}_{j}\left(0\right)=0, j=1, 2,\end{array}$$

where ${V}_{0}$ is the initial volume of proliferating cells. The initial conditions are chosen such that in the absence of treatment the tumor cells have strictly exponential growth. For a detailed derivation, see [28]. Combining Eqs. 1, 4, 5, 6, and 7 gives the complete set of equations describing proliferating and non-proliferating cells’ turnover. The total tumor volume is given by

8 $${V}_{tot} ={V}_{1}+{V}_{2}+{V}_{3}+{V}_{4}+{U}_{1}+{U}_{2}.$$

#### Radiosensitizing effect

The radiosensitizers investigated in this paper are all ATM inhibitors and thus, have similar mechanisms of action. Once a single or double-stranded DNA break has occurred due to radiation, the body will try to repair it [43]. The radiosensitizers stimulate the effect of the radiation by interfering with these repair mechanisms, as described in [3]. For the long-term radiation effect, this stimulation is modeled by scaling the accumulated radiation dose ${D}_{Acc}$ in the alternative expressions for inhibition in Eq. 2 by a factor $\left(1+b C\right)$,

9 $$\begin{array}{c}I\left({D}_{Acc}\right)={e}^{-\gamma {D}_{Acc}\left(1+b C\right)}\\ I\left({D}_{Acc}\right)=1-\frac{\gamma {D}_{Acc}\left(1+b C\right)}{I{D}_{50}+{D}_{Acc}\left(1+b C\right)}\\ I\left({D}_{Acc}\right)=1-\gamma {D}_{Acc}\left(1+b C\right)\end{array}$$

where *C* is the average or maximum plasma concentration of radiosensitizer after each radiation application. Both PK models and ${C}_{max}$ were evaluated to find best fit to data.

For the short-term radiation effect, the stimulating effect of the radiosensitizer is described by an increase in the proportion of tumor cells that are radiation damaged at each radiation application and the modified version of the linear-quadratic equation which accounts for this is,

10 $$F\left({D}_{R},C\right)=1-{e}^{-(1 + a C)(\alpha {D}_{R} + \beta { D}_{R}^{2})}$$

Parameters *a* and *b* are associated with the potency of each specific radiosensitizer.

### Tumor static exposure

Tumor Static Exposure (TSE) is a concept used for quantifying the efficacy of different combination therapies as well as predicting optimal dose schedules and ranking different compounds [26, 34, 35]. In this paper, we consider and compare both a simulation-based and an analytical method of finding the TSE.

#### Analytical solution for tumor static exposure

TSE is used to determine the necessary exposure of one or several treatments to keep the tumor in stasis. Mathematically this occurs when the derivative of the tumor volume is zero, i.e., we can define TSE for our model as all combinations of $C$ and ${D}_{Acc}$ such that

11 $$\frac{d{V}_{tot}\left(C,{D}_{Acc}\right)}{dt}=0.$$

**Fig. 2:**
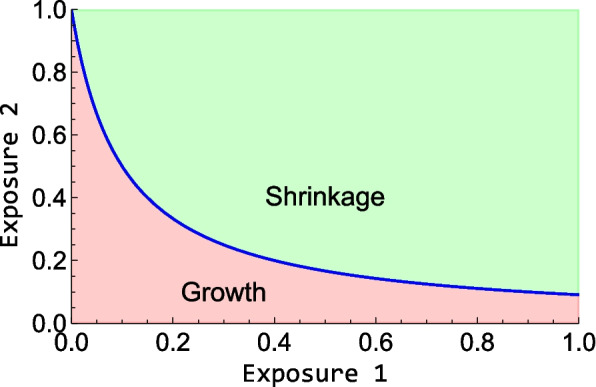
Tumor Static Exposure curve for two different exposures. Both axes can represent plasma concentration of an anticancer drug, one axis can also represent radiation dose. All exposure pairs on the blue line result in the tumor being in stasis and thus, exposures below it (red area) lead to tumor growth while exposures above it (green area) lead to tumor shrinkage. The values on the axes are only chosen for illustrative purposes

Here and henceforward the fractionation of the radiation treatment is assumed to be the same as in the 6 weeks studies. This results in a curve in a diagram with one axis representing drug plasma concentration and the other radiation dose, referred to as a TSE-curve. Administering doses yielding an exposure above the TSE-curve, the model predicts tumor regression and eventual eradication. An example of a TSE-curve is shown in Fig. 2.

To find an analytical expression for the TSE-curve, as in [35], some simplifying assumptions are made. Since all proliferating cells are in the ${V}_{1}$ compartment, it is sufficient that ${V}_{1}$ is in stasis for the tumor to reach stasis eventually. An analytical expression for turnover of proliferating cells, i.e., Eq. 1 combined with Eq. 4, leads to a complex expression that is hard to evaluate because of the treatment schedule. The short-term radiation effect only contributes to tumor regression during the treatment period, hence once the treatment stops, only the long-term effect will impact tumor growth. Thus, an analytical expression can be found by considering only the long-term radiation effect. We set the derivative of ${V}_{1}$ in Eq. 1 equal to zero. Using a linear inhibition function (last expressions in Eq. 2), one obtains the following expression for the TSE-curve,

12 $${D}_{Acc}=\frac{1-\frac{{k}_{k}}{{k}_{g}}}{\gamma \left(1+b C\right)}$$

Equation 12 describes the total radiation dose required for tumor stasis for different values of plasma concentration of the radiosensitizer C.

#### Simulation-based solution for tumor static exposure

The simplified approach outlined above neglects the short-term radiation effect. Therefore, we propose a simulation-based method for calculating the TSE-curve, which considers both the long-term and short-term treatment effects.

The calibrated tumor model can be used to simulate tumor volume over time, and can therefore be used to evaluate the derivative for different combinations of radiosensitizer exposure, *C,* and accumulated dose, ${D}_{Acc}$. A specific time point ${t}^{*}$ must be selected for the evaluation of the derivative and to construct a TSE-curve means finding combinations of *C* and ${D}_{Acc}$, which makes the derivative sufficiently small. This is done by solving the following optimization problem for a range of fixed values of ${D}_{Acc}$,

13 $$\begin{array}{c}\mathrm{minimize}\, C\\ \mathrm{subject\, to}\,\frac{d{V}_{tot}\left(C,{D}_{Acc}\right)}{dt}{|}_{t={t}^{*}}\le \epsilon \end{array}$$

where $\epsilon$ is a sufficiently small number. Thus, solving this optimization problem for different ${D}_{Acc}$, we obtain combination pairs (*C*, ${D}_{Acc}$) that all keep the tumor in stasis. The simulated TSE-curve depends on the selected time ${t}^{*}$. Since the short-term effect is only active during the treatment period, its contribution to the simulated TSE-curve is reduced for large ${t}^{*}$. Letting ${t}^{*}\to \infty$ leads to a TSE-curve based only on the long-term effect, i.e., the one in Eq. 12. We chose ${t}^{*}=60$ days and $\epsilon ={10}^{-4}$ based on simulations for a range of potential ${t}^{*}$ and $\epsilon$.

**Fig. 3:**
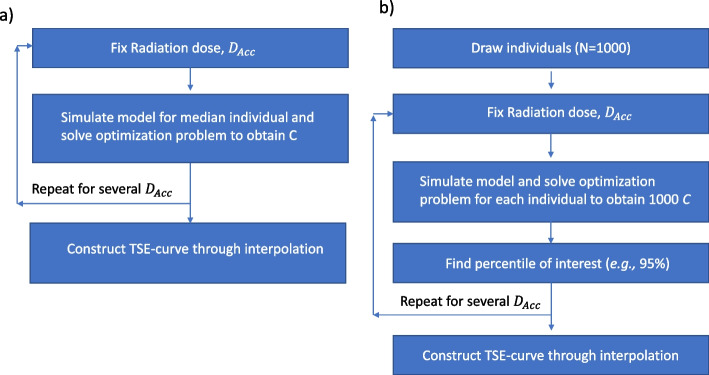
**a** The algorithm used to construct the median TSE-curve. The optimization problem formulated in Eq. 13 is iteratively solved to find the radiosensitizer exposure, which renders the median individual’s tumor to be in stasis for different radiation doses. The TSE-curve is then created through interpolation. **b** The algorithm used to construct percentile TSE-curves. A virtual dataset of individuals (here 1000) is first created. The optimization problem formulated in Eq. 13 is then iteratively solved for each individual, to find the radiosensitizer exposure that renders that individual’s tumor to be in stasis at different radiation doses. For a given percentile of interest, e.g., 95%, the radiosensitizer exposure that is sufficient for this percentile of tumors to be in stasis can be calculated for each radiation dose and the TSE-curve is then constructed through interpolation

Parameter estimations are performed using the NLME framework [44] and thus, some parameters in the model are defined by a population distribution. However, using the median parameters result in a simulated TSE-curve that describes the necessary exposure combinations for the median individual. We have developed an algorithm that computes this TSE-curve and it works in the following way. The model is first simulated for a fixed radiation dose and the optimization problem in Eq. 13 is solved to find the corresponding C such that the exposure pair (*C*, ${D}_{Acc}$) results in tumor stasis. The curve is then constructed by repeating this procedure for several different radiation doses The algorithm is shown in Fig. 3a.

We have also developed a similar algorithm that utilizes Monte Carlo simulations to predict exposure pairs necessary for a given percentile of tumors to be in stasis. The first step in this algorithm is to create a large set of virtual individuals (here 1000) by drawing parameter values from the population parameter distribution. Then, for a set of given radiation doses covering the explored range, the optimization problem is solved to find the radiosensitizer concentration leading to tumor stasis for each virtual individual. In this way, we compute an empirical distribution of *C*-values for a fixed ${D}_{Acc}$. A given percentile, e.g., 95%, is then extracted from this empirical distribution for each radiation dose and the TSE-curve for that percentile is drawn through interpolation. This algorithm is shown in Fig. 3b.

### Computational methods

Mathematical modeling and parameter estimation were performed using an NLME modeling approach based on the first-order conditional estimation (FOCE) method [45]. The computational framework used was developed at the Fraunhofer–Chalmers Research Centre for Industrial Mathematics (Gothenburg, Sweden) [46]. The tumor model was simultaneously fitted to tumor volume data from all treatment groups in all three studies. The quotient α/β was set to the typical value of 10 Gy [35]. The observation error model used included both a proportional and additive term. The model was validated based on the precision of estimated parameters, in terms of relative standard error (RSE), individual fit, empirical Bayes estimates (EBEs) [47], Akaike information criterion (AIC) [48], and visual predictive checks (VPC) [49]. Log-normal between-subject variability was accounted for in the parameters γ, α, and ${V}_{0}$ (no correlation assumed). Furthermore, there was also a difference in mean initial tumor volume between the studies, and thus the median of ${V}_{0}$ was allowed to vary between the studies. To avoid biased EBEs, the median of γ was also allowed to vary between the studies. Taking into consideration both the between-subject variability and between-study variability, the individual $\gamma$ value for individual j was represented by the following expression

14 $${\gamma }_{j}=\left({\gamma }_{1}\cdot Stud{y}_{1}+{\gamma }_{2}\cdot Stud{y}_{2}+{\gamma }_{3}\cdot Stud{y}_{3}\right){e}^{{\eta }_{j}}.$$

where $Stud{y}_{k}=1$ if individual *j* is in study *k* and 0 otherwise and ${\gamma }_{1}, {\gamma }_{2},$ and ${\gamma }_{3}$ are the specific median $\gamma$ value for each study [12, 13].

We also performed cross-validation by re-calibrating the model without the data from the 100 mg/kg $R{s}_{1}$ group in study 3 and then predicting the dynamics of this group by performing a VPC. Furthermore, we performed a sensitivity analysis around the point estimates at the time ${t}^{*}=60$ days to determine which parameters affect tumor volume the most. Sensitivities were calculated for each treatment group and normalized against the largest value within each group. Parameters of the same type, e.g., ${\gamma }_{1}$, ${\gamma }_{2}$, and ${\gamma }_{3}$ were lumped together as $\overline{\gamma }$ and the mean and standard deviation over all treatment groups affected by the parameter were estimated.

## Results

### Exposure to radiosensitizer

We found that using the observed average maximum concentration, ${C}_{max}$, to represent the exposure to the radiosensitizers provided the best fit to data. See Figure 7 in Appendix for a VPC comparing ${C}_{max}$ and average concentration. Moreover, none of the radiosensitizers were accumulated in the plasma as a result of the repeated dosing.${C}_{max}$ for each treatment group is shown in Table 2. Box whisker charts of the observed maximum concentration of the test compounds in the different treatment groups are also shown in Figure 8 in Appendix.

**Table 2:** Observed average maximum concentration for each treatment group

Test Compound	Dose [mg/kg]	${C}_{max}$(SD) [$\mathrm{\mu g}/\mathrm{mL}$]
Study 1		
$R{s}_{1}$	100	1 (0.5)
$R{s}_{2}$	25	2 (0.2)
$R{s}_{2}$	100	5 (1.2)
$R{s}_{3}$	20	7 (2.2)
Study 2		
$R{s}_{1}$	10	1 (0.4)
$R{s}_{1}$	50	2 (0.5)
$R{s}_{1}$	200	9 (2.9)
Study 3		
$R{s}_{1}$	25	5 (1.6)
$R{s}_{1}$	50	6 (4)
$R{s}_{1}$	100	7 (2.4)

### Pharmacodynamics

**Fig. 4:**
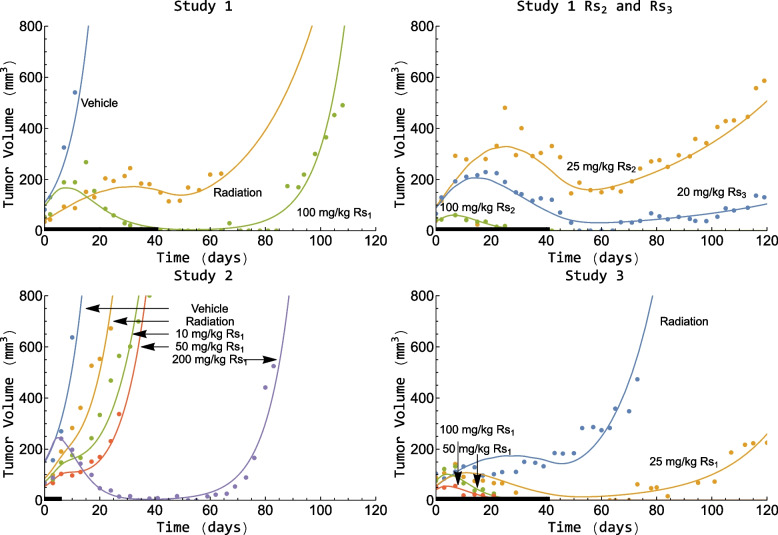
Tumor volume versus time for one individual per treatment group and study. The continuous lines are the model predictions, and the dots are the experimental observations. Radiosensitizer and/or radiation treatment were given 5 days a week for either 1 or 6 weeks, and the black line along the x-axis denotes the treatment period of each study

The model described the xenograft data from each treatment group of each study well. To illustrate this, examples of individual fits for each treatment group and study are shown in Fig. 4. The model was able to distinguish between regrowth and eradication for all tumors. The observed fractions of eradicated tumors in each treatment group, given radiosensitizer, and study are shown in Table 3.

**Table 3:** Observed fraction of eradicated tumors observed in each treatment group

Test Compound	Dose [mg/kg]	Observed fraction of eradicated tumors
Study 1		
$R{s}_{1}$	100	1/9
$R{s}_{2}$	25	9/9
$R{s}_{2}$	100	8/9
$R{s}_{3}$	20	6/9
Study 2		
$R{s}_{1}$	10	0/10
$R{s}_{1}$	50	1/10
$R{s}_{1}$	200	3/9^a^
Study 3		
$R{s}_{1}$	25	8/10
$R{s}_{1}$	50	8/9^a^
$R{s}_{1}$	100	10/10

^a^One early dropout not included

#### Parameter estimation

Based on AIC, it was determined that the linear inhibitory function gave the best fit to the data. The estimated parameters for the model are shown in Table 4 and VPCs for all treatments group are shown in Figures 10, 11 and 12 in Appendix. The system parameters were all estimated with reasonable precision (RSE < 25%), the parameter with the highest RSE was ${\gamma }_{2}$ with 20%. The test compound parameters, except for ${a}_{Rs1}$, were all estimated with precision above RSE 25%. The between-subject variability was high for all three parameters assumed to have a distribution in the population. To investigate why the RSE was over 25% for most of the test compound parameters, we simulated a new dataset for study 1 and $R{s}_{2}$ with artificially lowered between-subject variability and re-estimated the parameters (see Table 6 in Appendix). The result from the cross-validation is shown in Figure 13 in Appendix and as can be seen, the model was able to predict this treatment group well.

**Table 4:** Estimated pharmacodynamic parameters after fitting the radiation and radiosensitizing model to the data of studies 1, 2, and 3

Parameter	Population Median (RSE%)	Between-subject variability^a^ (RSE%)	Description
${k}_{g}$[1/day]	0.44 (1.3)	-	Natural growth rate
${k}_{k}$[1/day]	0.3 (1.7)	-	Natural kill rate
$\alpha$[1/Gy]	0.06 (7)	50 (25)	Linear radiation parameter
${\gamma }_{1}, {\gamma }_{2}, {\gamma }_{3}$[1/Gy]	0.027 (11), 0.044 (20), 0.02 (15)	64 (34)	Growth rate inhibition parameter
${V}_{\mathrm{0,1}}, {V}_{\mathrm{0,2}}, {V}_{\mathrm{0,3}}$[mm^3^]	26 (4.3), 44 (4.4), 22 (5)	40 (18)	Initial volume of proliferating cells
${a}_{Rs1}$[$\mathrm{mL}/\mathrm{\mu g}$]	0.65 (13)	-	Stimulation of short-term effect
${a}_{Rs2}$[$\mathrm{mL}/\mathrm{\mu g}$]	0.2 (30)	-	
${a}_{Rs3}$[$\mathrm{mL}/\mathrm{\mu g}$]	0.14 (32)	-	
${b}_{Rs1}$[$\mathrm{mL}/\mathrm{\mu g}$]	0.26 (31)	-	Stimulation of long-term effect
${b}_{Rs2}$[$\mathrm{mL}/\mathrm{\mu g}$]	0.4 (34)	-	
${b}_{Rs3}$[$\mathrm{mL}/\mathrm{\mu g}$]	0.16 (40)	-	
${\sigma }_{prop}$[%]	0.35 (2.2)	-	Proportional error
${\sigma }_{add}$[mm^3^]	4.4 (7.3)	-	Additive error

^a^Calculated as the square root of the diagonal entries of the population covariance matrix

Since there is a difference in median initial tumor volume in the three studies, inter-study variability was introduced to ${V}_{0}$. The estimated medians were 56 mm^3^ for study 1, 110 mm^3^ for study 2, and 66 mm^3^ for study 3, which was in accordance with the data. The estimated $\gamma$-values, inhibition of the growth rate after a total radiation dose of 60 Gy, corresponding to a 16%, 26%, and 13% inhibition, for ${\gamma }_{1}$, ${\gamma }_{2}$, and ${\gamma }_{3}$, respectively. These numbers represent the inhibition if radiation treatment is given alone. Concomitant radiation treatment with a radiosensitizer further reduces the growth rate. For example, for ${\gamma }_{1}$, $R{s}_{1}$ increased the inhibition of the growth rate to 20% and 55% at the lowest and highest dose, respectively. The estimate of $\alpha$ to 0.06 1/Gy, corresponds to 11% of the proliferating cells being lethally damaged each time the tumors are irradiated with 2 Gy. This fraction is increased to 17% and 55% at the lowest and highest dose of ${Rs}_{1}$. Using the estimate for the natural growth and kill rates, the doubling time for an untreated tumor was approximately 5 days. Table 5 shows the estimated growth rate from several other studies. The estimated growth rates range from 0.024 to 1.14 1/day, which compares well with our estimate of 0.14 1/day. The results from the sensitivity analysis are presented in Table 7 in Appendix. The two most important parameters were ${k}_{g}$ and ${k}_{k}.$

**Table 5:** Estimated growth rates for human xenografts of different cancer types in mice reported in the literature

${k}_{net}$[1/day]	${k}_{g}$[1/day]	${k}_{k}$[1/day]	Paper	Cancer Type
0.127	0.127	-	Koch et al. [27]	Colorectal
0.144	0.144	-	Goteti et al. [32]	Colorectal
0.27	0.27	-	Simeoni et al. [31]	Colorectal
0.166	0.166	-	Ouerdani et al. [50]	Kidney
0.024	0.024	-	Watanabe et al. [23]	Lung
0.05	0.14	0.09	Cardilin et al. [28]	Lung
0.14	0.44	0.3	Baaz et al	Lung
0.14	0.4	0.26	Cardilin et al. [35]	Lung
0.22	0.22	0.28	Cardilin et al. [37]	Lung
0.146	0.146	-	Simeoni et al. [31]	Ovarian
0.57696^a^	0.577^a^	3.86 (10−5)^a^	Gabrielsson et al. [26]	Leukemia
1.14	1.14	-	Miao et al. [33]	Pancreatic

- Not estimated

^a^Units [mm/day]

### Tumor static exposure

**Fig. 5:**
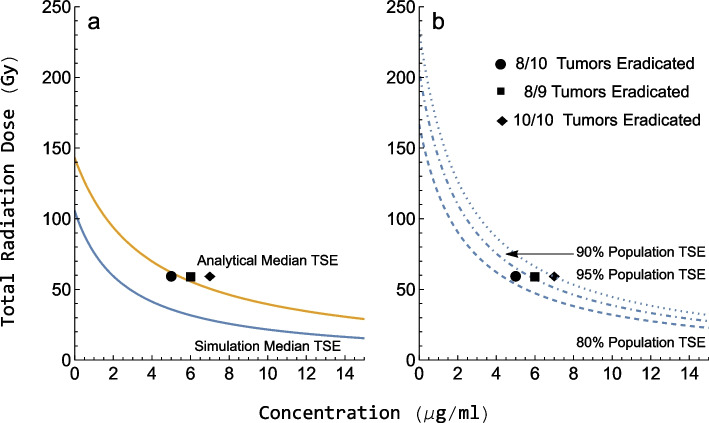
TSE curves where the total radiation dose is plotted against the concentration of radiosensitizer. Exposure pairs on the curves are predicted to result in tumor stasis for the given population percentile. **a** Median TSE predictions using the simulation-based method (blue) and the analytical long-term method (yellow). **b** 80%, 90%, and 95% percentile predictions using the simulation-based TSE. The three black markers in both subfigures represent the exposure combinations of three treatment groups in study 3. The corresponding number of eradicated tumors observed for different exposure combinations is shown in the legend

Using the parameters from Table 4, TSE-curves were created using both the simulation-based and the analytical long-term methods (Eq. 12). Figure 5a illustrates the difference between the two methods using the specific parameters from study 3. The exposure combinations required for tumor stasis are predicted to be lower using the simulation-based method compared to using the long-term analytical method. However, the radiosensitizer concentrations predicted to induce tumor stasis in only half of the population with the analytical method led to the eradication of most tumors in the three experimental studies. These experimental data are better captured by the simulation-based method, as shown in Fig. 5b. The simulated TSE-curve for the 80%, 90%, and 95% population percentiles agree well with the results of the efficacy studies, in which respectively 8 out of 10, 8 out of 9, and 10 out of 10 tumors were eradicated with the treatments.

**Fig. 6:**
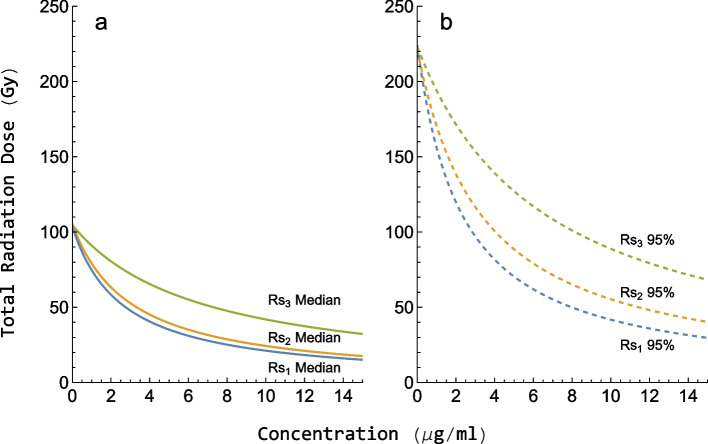
Median (**a**) and 95% population TSE-curves (**b**) for each of the three radiosensitizers as continuous and dashed lines, respectively. $R{s}_{1}$, $R{s}_{2}$, and $R{s}_{3}$ are shown in blue, yellow, and green, respectively. The total radiation dose has been plotted versus the concentration of radiosensitizer. Exposure pairs on the curves are predicted to result in stasis for **a** 50% and **b** 95% of the tumors in a population. The ranking of the radiosensitizers is based on this figure

To rank the radiosensitizers, inter-study variability was accounted for by choosing the smallest $\gamma$-value, i.e., ${\gamma }_{3}$, leading to a more conservative estimation of the TSE-curve. Radiation treatment without radiosensitizer was predicted to require a total radiation dose of 220 Gy for 95% of the tumors to be eradicated. The TSE-curves, shown in Fig. 6, predict that this dose can be reduced to 50 Gy, 65 Gy, and 100 Gy for $R{s}_{1}$, $R{s}_{2}$, and $R{s}_{3}$, respectively, at a ${C}_{max}$ of 8 $\mathrm{\mu g}/\mathrm{mL}$.

## Discussion

This paper focuses on ranking radiosensitizers based on their anticancer efficacy. To accomplish this, a radiation and radiosensitizer model was calibrated to human tumor xenograft data, and a simulation-based TSE method was developed and used to rank three radiosensitizers based on their ability to induce tumor stasis and eradication.

### Exposure to radiosensitizer

**Fig. 7:**
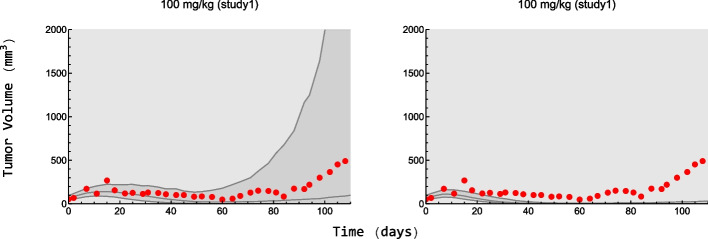
VPCs for the 100 mg/kg $R{s}_{1}$ using ${C}_{max}$ (left) and average radiosensitizer concentration (right). The continuous line in the middle of the grey area is the simulated median, and the grey area is a 90% confidence interval for the median. The red dots are the observed median from the experimental data. Tumor volume is shown on the y-axis and time on the x-axis

It has previously been explored whether the maximum or the average plasma concentration is the better predictor of anticancer efficacy [29]. We tested both measures and found that the maximum plasma concentration of the radiosensitizers was best to drive the radiosensitizing effect in the pharmacodynamic model (see Figure 7 in Appendix). We also investigated using ${C}_{max}$ and one-compartment pharmacokinetic models to drive the pharmacodynamics and found that the ${C}_{max}$ approach gave a better model fit, likely because only the concentration at the time of irradiation impacts the dynamics.

Different test compound formulations of $R{s}_{1}$ can explain the seemingly large variation in exposure between studies 1 and 2/3. The variation in exposure between studies 2 and 3 could additionally be explained by the fact that different compound batches were used and due to experimental variability [12].

### Pharmacodynamics

Model-based assessment of data from combination therapy studies uses data effectively and yields a deeper understanding of the disease and/or treatment. In addition, modeling enables simulation-based analysis, potentially reducing the need for animal testing and thus improving animal welfare [51].

Our radiation and radiosensitizing model can describe xenograft data for different radiosensitizing agents and across multiple studies. Recently, it has also been used to model diffuse intrinsic pontine glioma xenograft data [52]. The model was able to distinguish between tumor eradication and regrowth for all individuals in all studies. Minor trends could be observed in the VPC for the 10 mg/kg $R{s}_{1}$ study 2 group, however, a large majority of the observed medians for this treatment group were still contained inside the 90% confidence region (see Figure 11 in Appendix).

#### Parameter estimation

The estimated growth rate compared well with what has been reported earlier, see Table 5. Moreover, other sources have estimated the $\alpha$ parameter in the linear quadratic model between 0.0055 and 0.41 1/Gy, similar to our estimate of 0.06 1/Gy [23, 24, 35, 53, 54]. The somewhat high RSE for some radiosensitizer parameters is likely due to the large between-subject variability in the corresponding radiation parameters $\alpha$ and $\gamma$, which makes it difficult to distinguish between test compound effect and population variability. We verified this by using the model to simulate a population with artificially reduced between-subject variability for study 1 (only $R{s}_{2}$ dose groups) and then re-estimating the model parameters to these data, which resulted in RSE below 25% for all drug parameters.

#### Inter-study and between-subject variability

Initially, inter-study variability was not included on any parameter, but this resulted in clear bias between studies in the EBEs for both $\gamma$ and ${V}_{0}$. Therefore, we introduce it to both of these parameters.

**Fig. 8:**
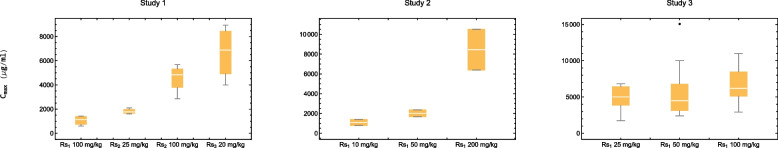
Boxplots showing the maximum observed plasma concentrations for each dose level of radiosensitizer in each study. Black horizontal lines represent the average maximum observed plasma concentration, which was used to drive the pharmacodynamics of the model

**Fig. 10:**
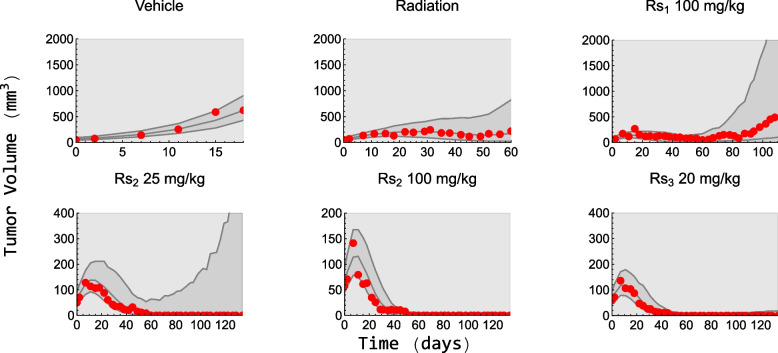
VPCs for the different treatment groups in study 1. The continuous line in the middle of the grey area is the simulated median, and the grey area is a 90% confidence interval for the median. The red dots are the observed median from the experimental data. Tumor volume is shown on the y-axis and time on the x-axis

**Fig. 11:**
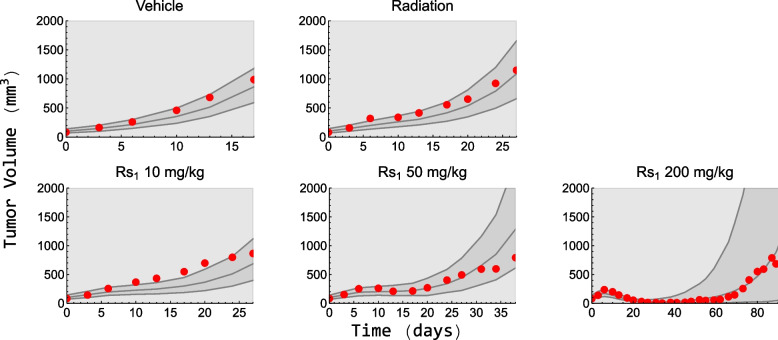
VPCs for the different treatment groups in study 2. The continuous line in the middle of the grey area is the simulated median, and the grey area is a 90% confidence interval for the median. The red dots are the observed median from the experimental data. Tumor volume is shown on the y-axis and time on the x-axis

**Fig. 12:**
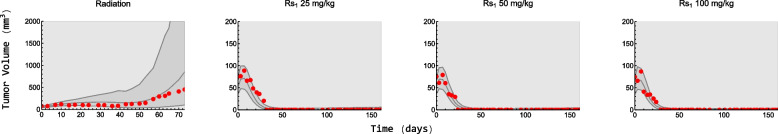
VPCs for the different treatment groups in study 3. The continuous line in the middle of the grey area is the simulated median, and the grey area is a 90% confidence interval for the median. The red dots are the observed median from the experimental data. Tumor volume is shown on the y-axis and time on the x-axis

**Fig. 13:**
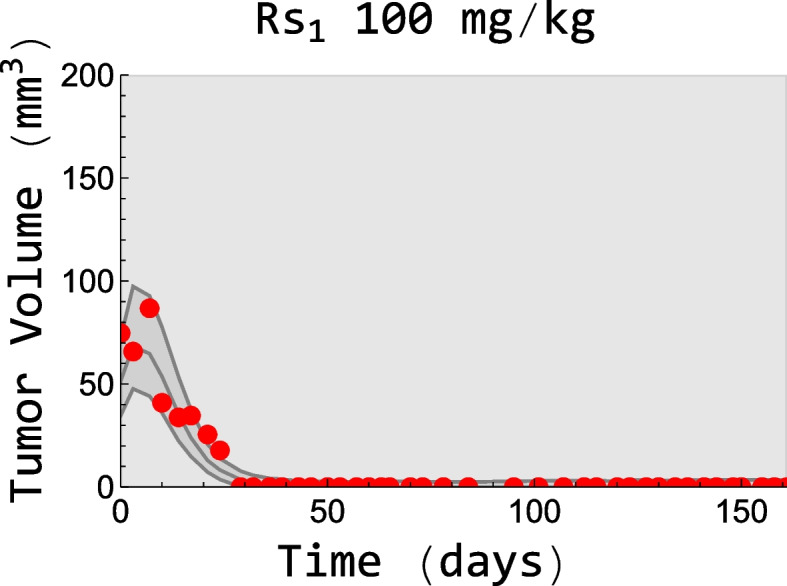
Cross-validation VPC for the 100 mg/kg $R{s}_{3}$ treatment group in study 3. The continuous line in the middle of the grey area is the simulated median, and the grey area is a 90% confidence interval for the median. The red dots are the observed median from the experimental data. Tumor volume is shown on the y-axis and time on the x-axis

The variability in $\gamma$ and $\alpha$ were slightly larger than the values reported in an earlier work [35], which is to be expected, given the larger dataset with multiple studies and radiosensitizers. Some shrinkage was observed in the EBEs for $\gamma$ (see Figure 8 in Appendix) in study 3, likely because most tumors in this study were eradicated early in the study. The median predictions were still in good agreement with the data (see Figure 11 in Appendix).

Two crucial issues in oncology are high attrition rates for compounds as they enter clinical trials and failure to reproduce published results [8–11]. Different reasons for these two issues are commonly cited, but one that they all share is the complexity of the disease [55–57]. Tumors and individuals are highly heterogeneous and therefore do not respond the same way [58]. Therefore, quantifying between-subject variability is essential particularly when such variability is large, as in the present studies. Moreover, quantification of between-subject variability allows for model predictions on a population level, thereby capturing the biological heterogeneity of the disease.

Another factor to consider to address the above issues is inter-study variability. Differences in pharmacodynamics or pharmacokinetics are often observed across studies and can help explain why a test compound that showed promise in preclinical studies failed in a clinical trial. In our study, the long-term radiation parameter $\gamma$ varies depending on the study used. Therefore, we have taken a conservative approach by using the parameters associated with the lowest efficacy for our predictions, to not overpredict the efficacy.

Although researchers have shown that preclinical efficacy is correlated with clinical efficacy, translating results from preclinical studies to a clinical setting is still an extremely challenging task [59–61]. This is also cited as one of the reasons for the high attrition rates of drugs tested in the clinic [62]. Therefore, the translational potential of the model we present here must be thoroughly investigated before it can be used to make clinical predictions. This could for example take the form of calibrating the model using clinical longitudinal data and comparing the parameter estimates to the ones we present in this paper. Performing such a study would hence give an idea of how to scale preclinically estimated parameters for clinical use. More comprehensive frameworks for performing preclinical to clinical translation, based on similar models and data as we use in this paper, have recently been published and these could be interesting to apply to our model [63, 64].

### Tumor static exposure

TSE is a model-based tool to predict the efficacy of anticancer agents given as single agents or in combination [27, 28, 33, 34]. In this paper, we have used TSE to analyze and compare the effects of radiation with three different radiosensitizing compounds. Three different TSE-curves were generated to predict tumor shrinkage for different combinations of radiation dose and radiosensitizer concentration. An earlier work computed an approximate TSE-curve based only on the long-term effects of radiation and radiosensitizing treatment [35]. Using this TSE-curve comes with two potential problems, the first being that this simplification is only valid when the dominating effect is the long-term effect. The other problem is that the potency of each radiosensitizer is described by two parameters, *a* and *b*, but only the *b* parameter is present in Eq. 12. In this paper, we propose a simulation-based method for calculating the TSE-curve, which takes into consideration both the long-term and short-term treatment effects.

We see in Fig. 5a, that the earlier method for calculating the TSE-curve results in an overprediction of the exposure levels necessary for the median tumor to be eradicated compared with experimental data. In contrast, Fig. 5b shows that the simulation-based approach gives predictions that are in excellent agreement with observed tumor eradication for each treatment group. Thus, highlighting the importance of considering both short-term and long-term effects.

To develop efficacious anticancer drugs, there is a need for preclinical tumor models that better predict how well a test compound would perform in a clinical setting [55, 56]. To accomplish this, phenomena of growing complexity, such as drug/radioresistance, DNA repair, and long-term treatment effects, have been incorporated into preclinical tumor models [35, 40, 54, 65]. This increased model complexity makes it hard to compute, e.g., TSE analytically. Thus, the simulation-based method presented here becomes more suitable for complex tumor models where compounds may have more than one effect (e.g., act on more than one model compartment).

Moreover, when analytical equations are derived for the TSE (see, e.g., [28, 34, 37, 66]), the resulting predictions are generally valid only for a given treatment schedule (with varying dose levels). The simulation-based method allows for predictions using different treatment schedules, although great caution should be taken when extrapolating far from the schedules used in the underlying studies. This gives the simulation-based method further benefit as finding optimal treatment schedules is an integral part of mathematical oncology [19–21].

### Ranking of radiosensitizers

Our analysis and comparison of the three radiosensitizing agents are based on efficacy and do not account for toxicological effects. Moreover, since the exposure levels associated with the highest dose of all three radiosensitizers were similar, we implicitly assume that the toxicological profiles of the three compounds are similar. Thus, we only rank the three radiosensitizers based on their anticancer efficacy and we found $R{s}_{1}$ to perform best of the three candidate compounds.

Developing a toxicological model is still essential for determining the potential of a test compound [36]. Toxicological models can take different forms, e.g., a model that describes the decrease in absolute neutrophil count and platelet count due to chemotherapy has previously been developed [67]. Another type of toxicological model is one that determines the probability that a dose-limiting toxicity event occurs at different concentration levels [29, 68].

Though we have quantified the variability in the data, certain sources of error and uncertainty remain in our predictions. For example, the drugs and radiation treatment were only tested at specific dose levels and treatment schedules, and thus to make predictions outside these specific regimens requires extrapolation. In addition, the model parameters are estimated with uncertainty (measured in RSE) and this could potentially also be incorporated into the predictions to give a confidence interval for the predictions. However, we did not do this in this paper since the most important parameters, found through the sensitivity analysis, were estimated with such high precision.

## Conclusions

The radiation and radiosensitizer model was able to describe data from multiple studies and different radiosensitizing agents. Moreover, a cross-validation was performed to investigate the model’s predictive capabilities and the median behavior of the treatment group left out was successfully captured by the model. Both inter-study variability and between-subject variability were quantified using the NLME framework and the estimated model parameters compare well with values previously reported in the literature. A sensitivity analysis showed that the most important model parameters for predicting tumor eradication were estimated with especially high precision.

The simulation-based method of calculating the TSE-curve for the radiation and radiosensitizer model was shown to provide better tumor eradication predictions than an earlier version that only considered the long-term effect. The resulting TSE-curves from the simulated-based method better agreed with the observed number of eradicated tumors and may provide a more accurate ranking of radiosensitizers. This demonstrates the importance of using the simulation-based method for predicting the efficacy or ranking novel radiosensitizers. Furthermore, the simulation-based TSE was shown to be a generalization of the analytical TSE, i.e., the analytical method for computing TSE [35] can be considered a special case of the simulation-based TSE introduced in this paper. A crucial aspect that has not yet been accounted for is the translational potential of the model. TSE predictions may be particularly useful here since clinical studies commonly report treatment progress in terms of tumor growth, shrinkage, or stable disease [15].

The three radiosensitizers analyzed in this paper were ranked based on their anticancer efficacy and $R{s}_{1}$ was found to be the best, with $R{s}_{2}$ and $R{s}_{3}$ in second and third place. Including inter-study variability in the analysis was shown to be crucial, as excluding it may result in overprediction of anticancer efficacy. Furthermore, investigating inter-study variability for different treatments can also lead to better clinical predictions, e.g., recommended phase I/II doses.

## Figures and Tables

**Fig. 9:**
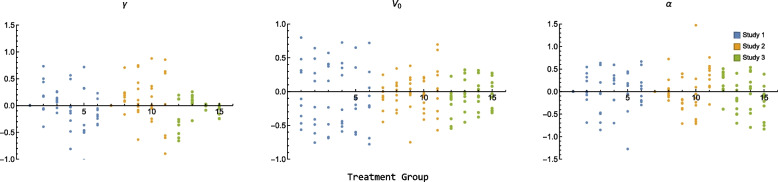
EBEs for the three log-normally distributed parameters. The different colors signify which study the different treatment groups are from, and the placement on the x-axis signifies which treatment group it is. The order the treatment groups are placed in is vehicle, radiation and then combination groups in increasing dose order. The different radiosensitizers in study 1 are placed in numerical order

## Data Availability

Due to its proprietary nature, raw data cannot be made publicly available. Requests made to the corresponding author will be forwarded to the appropriate persons for consideration.

## Appendix

**Table 6:** Estimated pharmacodynamic parameters after fitting the radiation and radiosensitizing model to simulate data with artificially lowered variance for study 1 (only dose groups for $R{s}_{2}$ were used)

Parameter	Population Median (RSE%)	Between-subject variability^a^ (RSE%)	Description
${k}_{g}$[1/day]	0.45 (3.88)	-	Natural growth rate
${k}_{k}$[1/day]	0.3 (5.5)	-	Natural kill rate
$\alpha$[1/Gy]	0.07 (4)	10 (8)	Linear radiation parameter
${\gamma }_{1}$[1/Gy]	0.04 (4)	10 (2)	Growth rate inhibition parameter
${V}_{\mathrm{0,1}}$[mm^3^]	26 (5)	39(14)	The initial volume of proliferating cells
${a}_{R{s}_{2}}$[$\mathrm{mL}/\mathrm{\mu g}$]	0.13 (16)	-	Stimulation of short-term effect
${b}_{R{s}_{2}}$[$\mathrm{mL}/\mathrm{\mu g}$]	0.3 (15)	-	Stimulation of long-term effect
${\sigma }_{prop}$[%]	0.23	-	Proportional error
${\sigma }_{add}$[mm^3^]	8.7	-	Additive error

^a^Calculated as the square root of the diagonal entries of the population covariance matrix

**Table 7:** Mean normalized sensitivity of the tumor volume at time ${t}^{*}$ for each type of model parameter. Parameters of the same type, e.g.*,*${\gamma }_{1}$, ${\gamma }_{2}$, and ${\gamma }_{3}$ were analyzed together as $\overline{\gamma }$

	Mean normalized sensitivity (SD)
${k}_{g}$	0.87 (0.25)
${k}_{k}$	-0.84 (0.13)
$\alpha$	-0.2 (0.13)
$\overline{\gamma }$	-0.11 (0.08)
$\overline{{v }_{0}}$	0.05 (0.01)
$\overline{a }$	-0.14 (0.09)
$\overline{b }$	-0.06 (0.05)

## Abbreviations

AIC: Akaike information criterion
ATM: Ataxia telangiectasia-mutated
EBE: Empirical Bayes estimate
FOCE: First-order conditional expectation
IR: Irradiation
NLME: Nonlinear mixed effects
PD: Pharmacodynamics
PK: Pharmacokinetics
RSE: Relative standard error
TSE: Tumor static exposure
VPC: Visual predictive check

## References

[1] Philips T, Hoppe R, Roach M. Leibel and Phillips Textbook of Radiation Oncology. 3rd ed. 2010.

[2] Harrison L, Chadha M, Hill R, Hu K, Shasha D (2002). Impact of tumor hypoxia and anemia on radiation therapy outcomes. Oncologist.

[3] Zimmermann A, Zenke FT, Chiu LY, Dahmen H, Pehl U, Fuchss T (2022). A new class of selective ATM inhibitors as combination partners of DNA double-strand break inducing cancer therapies. Mol Cancer Ther.

[4] Li Y, Cucinotta FA (2020). Mathematical model of ATM activation and chromatin relaxation by ionizing radiation. Int J Mol Sci.

[5] Wang H, Mu X, He H, Zhang XD (2018). Cancer radiosensitizers. Trends Pharmacol Sci.

[6] Her S, Jaffray DA, Allen C (2017). Gold nanoparticles for applications in cancer radiotherapy: mechanisms and recent advancements. Adv Drug Deliv Rev.

[7] Mi Y, Shao Z, Vang J, Kaidar-Person O, Wang AZ (2016). Application of nanotechnology to cancer radiotherapy. Cancer Nano.

[8] Arrowsmith J, Miller P (2013). Phase II and phase III attrition rates 2011–2012. Nat Rev Drug Discov.

[9] Mobley A, Linder SK, Braeuer R, Ellis LM, Zwelling L (2013). A survey on data reproducibility in cancer research provides insights into our limited ability to translate findings from the laboratory to the clinic. PLoS One.

[10] Ioannidis JPA (2005). Contradicted and initially stronger effects in highly cited clinical research. JAMA.

[11] Begley CG (2013). Six red flags for suspect work. Nature.

[12] Laporte-Simitsidis S, Girard P, Mismetti P, Chabaud S, Decousus H, Boissel JP (2000). Inter-study variability in population pharmacokinetic meta-analysis: when and how to estimate it?. J Pharm Sci.

[13] Andersson R, Kroon T, Almquist J, Jirstrand M, Oakes ND, Evans ND (2017). Modeling of free fatty acid dynamics: insulin and nicotinic acid resistance under acute and chronic treatments. J Pharmacokinet Pharmacodyn.

[14] Zhang P, Brusic V (2014). Mathematical modeling for novel cancer drug discovery and development. Expert Opin Drug Discov.

[15] Lombard A, Mistry H, Chapman SC, Gueoguieva I, Aarons L, Ogungbenro K (2020). Impact of tumour size measurement inter-operator variability on model-based drug effect evaluation. Cancer Chemother Pharmacol.

[16] Koga Y, Ochiai A (2019). Systematic review of patient-derived xenograft models for preclinical studies of anti-cancer drugs in solid tumors. Cells.

[17] Francisco JD, Peter RM, Anuradha R, Byron T, Ashleigh P, Jessica H (2013). Compound ranking based on a new mathematical measure of effectiveness using time course data from cell-based assays. Comb Chem High Throughput Screen.

[18] Paracha N, Reyes A, Diéras V, Krop I, Pivot X, Urruticoechea A (2020). Evaluating the clinical effectiveness and safety of various HER2-targeted regimens after prior taxane/trastuzumab in patients with previously treated, unresectable, or metastatic HER2-positive breast cancer: a systematic review and network meta-analysis. Breast Cancer Res Treat.

[19] Stein S, Zhao R, Haeno H, Vivanco I, Michor F (2018). Mathematical modeling identifies optimum lapatinib dosing schedules for the treatment of glioblastoma patients. PLoS Comput Biol.

[20] Pierrillas PB, Fouliard S, Chenel M, Hooker AC, Friberg LF, Karlsson MO (2018). Model-based adaptive optimal design (MBAOD) improves combination dose finding designs: an example in oncology. AAPS J.

[21] Gorissen BL, Unkelbach J, Bortfeld TR (2016). Mathematical optimization of treatment schedules. Int J Radiat Oncol Biol Phys.

[22] Vakil V, Trappe W (2019). Drug combinations: mathematical modeling and networking methods. Pharmaceutics.

[23] Watanabe Y, Dahlman EL, Leder KZ, Hui SK (2016). A mathematical model of tumor growth and its response to single irradiation. Theor Biol Med Model.

[24] Tariq I, Humbert-Vidan L, Chen T, South CP, Ezhil V, Kirkby NF (2015). Mathematical modelling of tumour volume dynamics in response to stereotactic ablative radiotherapy for non-small cell lung cancer. Phys Med Biol.

[25] Ribba B, Kaloshi G, Peyre M, Ricard D, Calvez V, Tod M (2012). A tumor growth inhibition model for low-grade glioma treated with chemotherapy or radiotherapy. Clin Cancer Res.

[26] Gabrielsson J, Gibbons FD, Peletier LA (2016). Mixture dynamics: combination therapy in oncology. Eur J Pharm Sci.

[27] Koch G, Walz A, Lahu G, Schropp J (2009). Modeling of tumor growth and anticancer effects of combination therapy. J Pharmacokinet Pharmacodyn.

[28] Cardilin T, Almquist J, Jirstrand M, Sostelly A, Amendt C, El Bawab S (2017). Tumor static concentration curves in combination therapy. AAPS J.

[29] Bottino DC, Patel M, Kadakia E, Zhou J, Patel C, Neuwirth R (2019). Dose optimization for anticancer drug combinations: maximizing therapeutic index via clinical exposure-toxicity/preclinical exposure-efficacy modeling. Clin Cancer Res.

[30] Tosca EM, Gauderat G, Fouliard S, Burbridge M, Chenel M, Magni P. Modeling restoration of gefitinib efficacy by co-administration of MET inhibitors in an EGFR inhibitor-resistant NSCLC xenograft model: a tumor-in-host DEB-based approach. CPT Pharmacometrics Syst Pharmacol. 2021;10(11):1396–411. 10.1002/psp4.12710 PMC8592518 34708556

[31] Simeoni M, Magni P, Cammia C, Nicolao GD, Croci V, Pesenti E (2004). Predictive pharmacokinetic-pharmacodynamic modeling of tumor growth kinetics in xenograft models after administration of anticancer agents. Cancer Res.

[32] Goteti K, Garner CE, Utley L, Dai J, Ashwell S, Moustakas DT (2010). Preclinical pharmacokinetic/pharmacodynamic models to predict synergistic effects of co-administered anti-cancer agents. Cancer Chemother Pharmacol.

[33] Miao X, Koch G, Straubinger RM, Jusko WJ (2016). Pharmacodynamic modeling of combined chemotherapeutic effects predicts synergistic activity of gemcitabine and trabectedin in pancreatic cancer cells. Cancer Chemother Pharmacol.

[34] Jumbe NL, Xin Y, Leipold DD, Crocker L, Dugger D, Mai E (2010). Modeling the efficacy of trastuzumab-DM1, an antibody drug conjugate, in mice. J Pharmacokinet Pharmacodyn.

[35] Cardilin T, Almquist J, Jirstrand M, Zimmermann A, Lignet F, El Bawab S (2019). Modeling long-term tumor growth and kill after combinations of radiation and radiosensitizing agents. Cancer Chemother Pharmacol.

[36] Cardilin T, Almquist J, Jirstrand M, Zimmermann A, Lignet F, El Bawab S (2022). Exposure-response modeling improves selection of radiation and radiosensitizer combinations. J Pharmacokinet Pharmacodyn.

[37] Cardilin T, Almquist J, Jirstrand M, Zimmermann A, Bawab SE, Gabrielsson J (2018). Model-based evaluation of radiation and radiosensitizing agents in oncology. CPT Pharmacometrics Syst Pharmacol.

[38] Simeoni M, Magni P, Cammia C, Nicolao GD, Croci V, Pesenti E (2004). Predictive pharmacokinetic-pharmacodynamic modeling of tumor growth kinetics in xenograft models after administration of anticancer agents. Can Res.

[39] Barcellos-Hoff MH, Park C, Wright EG (2005). Radiation and the microenvironment – tumorigenesis and therapy. Nat Rev Cancer.

[40] Sachs RK, Hlatky LR, Hahnfeldt P (2001). Simple ODE models of tumor growth and anti-angiogenic or radiation treatment. Math Comput Model.

[41] Eriksson D, Stigbrand T (2010). Radiation-induced cell death mechanisms. Tumor Biol.

[42] Forrester HB, Albright N, Ling CC, Dewey WC (2000). Computerized video time-lapse analysis of apoptosis of REC: Myc cells X-irradiated in different phases of the cell cycle. Radiat Res.

[43] Baskar R, Dai J, Wenlong N, Yeo R, Yeoh KW (2014). Biological response of cancer cells to radiation treatment. Front Mol Biosci.

[44] Leander J, Almquist J, Johnning A, Larsson J, Jirstrand M. NLMEModeling: a Wolfram Mathematica package for nonlinear mixed effects modeling of dynamical systems. arXiv:201106879 [stat]. 2020.

[45] Wang Y (2007). Derivation of various NONMEM estimation methods. J Pharmacokinet Pharmacodyn.

[46] Leander J, Almquist J, Johnning A, Larsson J, Jirstrand M. Nonlinear mixed effects modeling of deterministic and stochastic dynamical systems in Wolfram Mathematica. IFAC-Pap 2021;54:409–14

[47] Efron B (2014). Two modeling strategies for empirical Bayes estimation. Stat Sci.

[48] Akaike H (1974). A new look at the statistical model identification. IEEE Trans Autom Control.

[49] Bergstrand M, Hooker AC, Wallin JE, Karlsson MO (2011). Prediction-corrected visual predictive checks for diagnosing nonlinear mixed-effects models. AAPS J.

[50] Ouerdani A, Struemper H, Suttle AB, Ouellet D, Ribba B (2015). Preclinical modeling of tumor growth and angiogenesis inhibition to describe pazopanib clinical effects in renal cell carcinoma. CPT Pharmacometrics Syst Pharmacol.

[51] Fenwick N, Griffin G, Gauthier C (2009). The welfare of animals used in science: how the “Three Rs” ethic guides improvements. Can Vet J.

[52] Husband HR, Campagne O, He C, Zhu X, Bianski BM, Baker SJ (2021). Model-based evaluation of image-guided fractionated whole-brain radiation therapy in pediatric diffuse intrinsic pontine glioma xenografts. CPT Pharmacometrics Syst Pharmacol.

[53] Dhawan A, Kaveh K, Kohandel M, Sivaloganathan S (2014). Stochastic model for tumor control probability: effects of cell cycle and (a)symmetric proliferation. Theor Biol Med Model.

[54] Forouzannia F, Enderling H, Kohandel M (2018). Mathematical modeling of the effects of tumor heterogeneity on the efficiency of radiation treatment schedule. Bull Math Biol.

[55] Begley CG, Ellis LM (2012). Raise standards for preclinical cancer research. Nature.

[56] Ebos JML, Kerbel RS (2011). Antiangiogenic therapy: impact on invasion, disease progression, and metastasis. Nat Rev Clin Oncol.

[57] Komlodi-Pasztor E, Sackett DL, Fojo AT (2012). Inhibitors targeting mitosis: tales of how great drugs against a promising target were brought down by a flawed rationale. Clin Cancer Res.

[58] Moding EJ, Kastan MB, Kirsch DG (2013). Strategies for optimizing the response of cancer and normal tissues to radiation. Nat Rev Drug Discov.

[59] Wong H, Choo EF, Alicke B, Ding X, La H, McNamara E (2012). Antitumor activity of targeted and cytotoxic agents in murine subcutaneous tumor models correlates with clinical response. Clin Cancer Res.

[60] Gao H, Korn JM, Ferretti S, Monahan JE, Wang Y, Singh M (2015). High-throughput screening using patient-derived tumor xenografts to predict clinical trial drug response. Nat Med.

[61] Owonikoko TK, Zhang G, Kim HS, Stinson RM, Bechara R, Zhang C (2016). Patient-derived xenografts faithfully replicated clinical outcome in a phase II co-clinical trial of arsenic trioxide in relapsed small cell lung cancer. J Transl Med.

[62] Kola I, Landis J (2004). Can the pharmaceutical industry reduce attrition rates?. Nat Rev Drug Discov.

[63] Betts A, Clark T, Jasper P, Tolsma J, van der Graaf PH, Graziani EI (2020). Use of translational modeling and simulation for quantitative comparison of PF-06804103, a new generation HER2 ADC, with Trastuzumab-DM1. J Pharmacokinet Pharmacodyn.

[64] Xiong W, Friese-Hamim M, Johne A, Stroh C, Klevesath M, Falchook GS (2021). Translational pharmacokinetic-pharmacodynamic modeling of preclinical and clinical data of the oral MET inhibitor tepotinib to determine the recommended phase II dose. CPT Pharmacometrics Syst Pharmacol.

[65] Claret L, Girard P, Hoff PM, Van Cutsem E, Zuideveld KP, Jorga K (2009). Model-based prediction of phase III overall survival in colorectal cancer on the basis of phase II tumor dynamics. JCO.

[66] Xiang H, Reyes AE, Eppler S, Kelley S, Damico-Beyer LA (2013). Death receptor 5 agonistic antibody PRO95780: preclinical pharmacokinetics and concentration–effect relationship support clinical dose and regimen selection. Cancer Chemother Pharmacol.

[67] Iliadis A, Barbolosi D (2000). Optimizing drug regimens in cancer chemotherapy by an efficacy-toxicity mathematical model. Comput Biomed Res.

[68] Meille C, Gentet JC, Barbolosi D, André N, Doz F, Iliadis A (2008). New adaptive method for phase I trials in oncology. Clin Pharmacol Ther.

